# 

**Published:** 1879-07

**Authors:** 


					﻿ÇVhole Number,
ccccx.
July, 1879.
Number 1,
Vol. XXXIX.
THE
CHICAGO
MEDICÆL
J ournal and Examiner
EDITORS:
WILLIAM H. BYFORD, A.M./M.D.,
JAS. NEVINS HYDE, A.M., M.D. N. S. DAVIS, A.M., M.D.
DANIEL R. BROWER, M.D.
CHICAGO:
PUBLISHED BY THE CHICAGO MEDICAL PRESS ASSOCIATION.
188 Clark Street.
Read the Notice of this Journal among Advertisements.
				

